# Individualized prediction of chronic kidney disease for the elderly in longevity areas in China: Machine learning approaches

**DOI:** 10.3389/fpubh.2022.998549

**Published:** 2022-10-21

**Authors:** Dai Su, Xingyu Zhang, Kevin He, Yingchun Chen, Nina Wu

**Affiliations:** ^1^Department of Health Management and Policy, School of Public Health, Capital Medical University, Beijing, China; ^2^Department of Systems, Populations, and Leadership, University of Michigan School of Nursing, Ann Arbor, MI, United States; ^3^Thomas E. Starzl Transplantation Institute, University of Pittsburgh Medical Center, Pittsburgh, PA, United States; ^4^Department of Biostatistics, University of Michigan School of Public Health, Ann Arbor, MI, United States; ^5^Department of Health Management, School of Medicine and Health Management, Tongji Medical College, Huazhong University of Science and Technology, Wuhan, China; ^6^Research Center for Rural Health Services, Hubei Province Key Research Institute of Humanities and Social Sciences, Wuhan, China

**Keywords:** prediction, chronic kidney disease, elderly, machine learning, longevity areas, China

## Abstract

**Background:**

Chronic kidney disease (CKD) has become a major public health problem worldwide and has caused a huge social and economic burden, especially in developing countries. No previous study has used machine learning (ML) methods combined with longitudinal data to predict the risk of CKD development in 2 years amongst the elderly in China.

**Methods:**

This study was based on the panel data of 925 elderly individuals in the 2012 baseline survey and 2014 follow-up survey of the Healthy Aging and Biomarkers Cohort Study (HABCS) database. Six ML models, logistic regression (LR), lasso regression, random forests (RF), gradient-boosted decision tree (GBDT), support vector machine (SVM), and deep neural network (DNN), were developed to predict the probability of CKD amongst the elderly in 2 years (the year of 2014). The decision curve analysis (DCA) provided a range of threshold probability of the outcome and the net benefit of each ML model.

**Results:**

Amongst the 925 elderly in the HABCS 2014 survey, 289 (18.8%) had CKD. Compared with the other models, LR, lasso regression, RF, GBDT, and DNN had no statistical significance of the area under the receiver operating curve (AUC) value (>0.7), and SVM exhibited the lowest predictive performance (AUC = 0.633, *p*-value = 0.057). DNN had the highest positive predictive value (PPV) (0.328), whereas LR had the lowest (0.287). DCA results indicated that within the threshold ranges of ~0–0.03 and 0.37–0.40, the net benefit of GBDT was the largest. Within the threshold ranges of ~0.03–0.10 and 0.26–0.30, the net benefit of RF was the largest. Age was the most important predictor variable in the RF and GBDT models. Blood urea nitrogen, serum albumin, uric acid, body mass index (BMI), marital status, activities of daily living (ADL)/instrumental activities of daily living (IADL) and gender were crucial in predicting CKD in the elderly.

**Conclusion:**

The ML model could successfully capture the linear and nonlinear relationships of risk factors for CKD in the elderly. The decision support system based on the predictive model in this research can help medical staff detect and intervene in the health of the elderly early.

## Introduction

Chronic kidney disease (CKD) has become a major public health problem worldwide and has caused a huge social and economic burden, especially in developing countries (1). In 2017, the number of CKD patients worldwide reached 697.5 million, amongst which nearly one-sixth (132 million) were in China (2). CKD plays an important role in the development of end-stage renal disease (ESRD) (3), all-cause mortality (4), non-vascular health outcomes (5) and hospitalisations (6). The prevalence of CKD increases with age, and this problem is exacerbated by the aging of the Chinese population. The 2015 Annual Data Report of the China Kidney Disease Network showed that nearly half of CKD patients in China are over 60 years old (7). A study reported that the prevalence of CKD (Stages III–IV) in men and women between 55 and 64 years old is 6.1 and 13.1%, respectively; in the corresponding population of 75–84 years old, the prevalence of CKD is increased to 33.2 and 41.7% (8). In addition, the average age of patients with ESRD in China is 59 years; the patients with ESRD in China are younger than those in the USA (62.8 years) and Japan (64.7 years) (9). Therefore, the early identification, intervention and establishment of effective treatment strategies for potential Chinese CKD patients are crucial in controlling the number of Chinese CKD cases. The elderly population is a high-risk group in terms of CKD. Close attention must therefore be devoted to the elderly population.

Several studies have analyzed the association between CKD and its risk factors, which mainly include the following types of indicators: (1) demographic characteristics, such as sex (10), age (11), and marital status (12); (2) unhealthy lifestyle, including smoking (13), and alcohol consumption (14); (3) mental and physical health, including instrumental activities of daily living (IADL) and activities of daily living (ADL) (15), cognition (16), depression (17), body mass index (BMI) (18), and waist circumference (19); (4) chronic diseases, such as hypertension (20), diabetes (21), heart disease (22), stroke and cerebrovascular diseases (23) and cancer (24); and (5) biology medical indicators, including serum albumin (25), blood urea nitrogen (21), total cholesterol (26), triglyceride (27), urea acid (28), and hemoglobin (29). However, most algorithms for predicting CKD are based on a small number of patients and clinical predictors, and their predictive accuracy is usually uncertain (21, 30–32). The main research method used at present is multiple linear regression. The multiple linear regression model is based on the least squares method and assumes that the variables are independent of one another. However, the relationship between dependent and independent variables is complex and nonlinear, with high-dimensional correlation (33–36). Therefore, the performance of this model in terms of sensitivity and specificity is insufficient for use in predicting CKD.

Machine learning (ML) techniques have many advantages, including robustness to parametric assumptions, high power and accuracy, capability to model nonlinear effects, availability of numerous well-developed algorithms and capability to model high-dimensional data (37). ML techniques can be used to design data-driven models and algorithms with predictive capabilities in an unpredictable manner to achieve good results. ML models have been widely used in medical and health fields to assess disease risks and provide information for the establishment of clinical decision support systems, including predicting disease outcome (38), recommending treatment methods (39), and personalized medicine (40). Therefore, ML technology is an effective means for the early diagnosis of CKD.

To the authors' knowledge, no previous study has used ML methods combined with longitudinal data to predict the risk of CKD amongst the elderly in China in 2 years. Therefore, this study utilizes survey data from the Healthy Aging and Biomarkers Cohort Study (HABCS) in China as a sample to predict CKD amongst the elderly in longevity areas in China by using six ML models.

## Materials and methods

### Study design and setting

**Figure 1:**
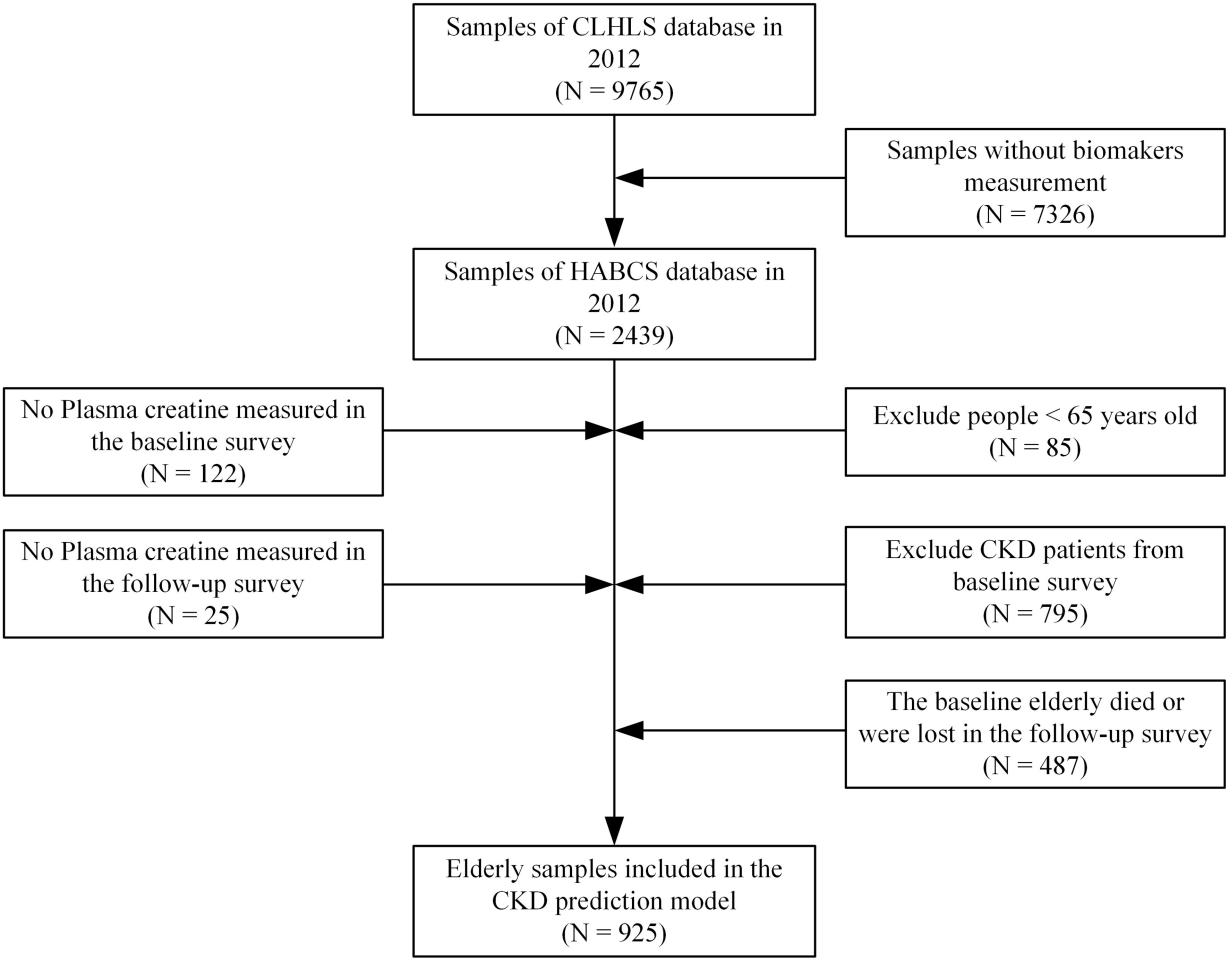
Study samples selection process.

The HABCS datasets were collected by the Center for Healthy Aging and Development Studies (CHADS) of National School of Development at Peking University and the Chinese Center for Disease Control and Prevention (CDC) from in-depth studies in the eight longevity areas in the Chinese Longitudinal Healthy Longevity Survey (CLHLS) 5th, 6th, and 7th waves in 2009, 2012, and 2014. We selected the surveyed individuals in 2012 as the baseline survey sample and the surveyed individuals in 2014 as the follow-up survey sample. The selected elderly did not have CKD in the baseline survey. The specific sample selection process is shown in Figure 1. The research conducted in this study was performed in accordance with the Declaration of Helsinki.

### Data collection

#### Questionnaire data

The CLHLS questionnaire data contained information on the research subjects' family structure, living arrangements and proximity to children, ADL, capacity for physical performance, self-rated health, self-evaluation of life satisfaction, cognitive functioning, chronic disease prevalence, care needs and costs, social activities, diet, smoking and drinking behaviors, psychological characteristics, economic resources and care giving and family support amongst elderly respondents and their relatives.

#### Laboratory examination

In this study, blood and urine samples of subjects corresponding to CLHLS samples in HABCS were collected for laboratory examination. Hemoglobin concentration (HC), which was amongst the indicators we collected for the laboratory examination, was measured on-site. The clinical test center of Capital Medical University used the Hitachi 7,180 automatic biochemical analyser produced in Japan and commercial diagnostic reagents produced by Roche Diagnostics company to examine other indicators, including plasma albumin, serum creatinine, blood urea nitrogen, blood urea acid, total cholesterol and triglyceride (41).

### Study variables

### Outcome variable

This study defined whether the sample in the follow-up survey met the diagnostic criterion of CKD as the outcome variable. When the sample met the diagnostic criterion of CKD, it was assigned a value of 1; otherwise, it was given a value of 0. The diagnostic criteria and definition for CKD were based on the Guidelines for the Screening, Diagnosis and Prevention of CKD published in China in 2017 and the Kidney Disease Outcomes Quality Initiative of the American Kidney Foundation (42). These two documents are very authoritative in China and the United States. The diagnostic criterion of CKD in these two documents was defined as estimated glomerular filtration rate (eGFR) <60 mL/min/1.73 m^2^. eGFR was calculated with the CKD-EPI equation, which is expressed as follows (43):


$$\begin{array}{lll} \text{eGFR} & = & 141\;\times \text{ min}\left(\text{Scr}/\kappa ,\;1\right)\alpha \;\times \text{ max}\left(\text{Scr}/\kappa ,\;1\right)-1.209\\ \; & \times & \;0.993\text{ Age }\times \;1.018\left(\text{if female}\right) \end{array}$$

where Scr is serum creatinine expressed in mg/dL, κ is 0.7 for females and 0.9 for males, α is −0.329 for females and −0.411 for males, min indicates the minimum of Scr/κ or 1 and max indicates the maximum of Scr/κ or 1.

### Predictors

We selected 22 variables of the respondents in the 2012 baseline survey as predictors of CKD, and these included the following: (1) demographic characteristics, such as gender (male vs. female), age (a continuous variable) and marital status (has a spouse vs. no spouse); (2) unhealthy lifestyle, including smoking (yes vs. no) and alcohol consumption (yes vs. no); (3) mental and physical health, including IADL/ADL (a continuous variable), cognitive function (a continuous variable), depression (yes vs. no), BMI (a continuous variable) and waist circumference (a continuous variable); (4) chronic diseases, such as hypertension (yes vs. no), diabetes (yes vs. no), heart disease (yes vs. no), stroke and cerebrovascular diseases (yes vs. no), cancer (yes vs. no), and blood disease (yes vs. no); and (5) biology medical indicators, including serum albumin (a continuous variable), blood urea nitrogen (a continuous variable), total cholesterol (a continuous variable), triglyceride (a continuous variable), urea acid (a continuous variable), and hemoglobin (a continuous variable).

In this study, we determined whether a respondent had hypertension based on whether the respondent was diagnosed with hypertension by a doctor. The diagnosis of Chinese doctors is based on the “China Guidelines for the Prevention and Treatment of Hypertension”, in which hypertension is defined as systolic blood pressure over 140 mmHg, diastolic blood pressure over 90 mmHg.

A modified version of Lawton's scale was used to measure impairments in IADL amongst the elderly samples (44). The scale includes the following self-reported activities: visiting neighbors, shopping, cooking a meal, washing clothes, walking continuously for 1 km at a time, lifting a weight of 5 kg, continuously crouching and standing up three times and taking public transportation. These items had three response categories, namely, “yes, independently”, “yes, but need some help” and “no, can't”; the three options were coded 3, 2 and 1, respectively. Impairment in ADL was measured using the Katz scale (45), which covers the following activities: bathing, dressing, going to the toilet, transferring indoors, continence and eating. The response categories of ADL were consistent with IADL's and coded similarly. We calculated the total score of 14 items as a respondent's final score, with a maximum of 42 points.

The measurement for depression used two levels of indicators, and an answer of “yes” to any question is considered a representation of depression (coded as 1, otherwise 0). The two questions were as follows: (1) Have you had a time in the last 12 months when you felt sad, blue or depressed for 2 weeks or more? (2) Have you had a time in the last 12 months lasting 2 weeks or more when you lost interest in most things, such as hobbies, work or activities, that you usually find pleasurable?

## Statistical analysis

In the training set (70% random sample), we developed different ML models to predict the probability of CKD for the elderly. The data structure of this study contains both predictor variables and outcome variables, so the ML prediction models were based on supervised learning algorithm. Meanwhile, the outcome variable is a binary variable, so this study focused on the classification problem, so as to solve the identification of CKD in the elderly. Therefore, we have constructed six ML classification algorithms based on supervised learning, logistic regression (LR), LR with lasso regularization (lasso regression), random forest (RF), gradient-boosted decision tree (GBDT), support vector machine (SVM) and deep neural network (DNN). A systematic comparison of the strengths and weaknesses of six machine learning algorithms was shown in Table 1 (46).

**Table 1:** A systematic comparison of the strengths and weaknesses of six machine learning algorithms.

**Supervised ML^a^ algorithms**	**Strengths**	**Weaknesses**
LR^b^	(1) Easier to implement, interpret.	(1) It tends to underperform when there are multiple or non-linear decision boundaries.
	(2) It makes no assumptions about distributions of classes in feature space.	(2) It is not flexible enough to naturally capture more complex relationships. DNN can easily outperform this algorithm.
	(3) It has a nice probabilistic interpretation of model parameters	(3) It requires average or no multicollinearity between independent variables.
	(4) It can interpret model coefficients as indicators of feature importance.	(4) Unless multinomial, generic LR can only classify variables that have two states (i.e., dichotomous).
Lasso regression	(1) It can be regularized to avoid overfitting.	(1) It leads to dimensionality reduction, which means the model is built using a lower dimensional dataset. This generally leads to a high bias errror.
	(2) Lower dimensional dataset is computationally efficient.	(2) It could be computationally expensive.
	(3) Outputs have a nice probabilistic interpretation.	(3) A poor value for hyperparameter might make the model performance worse.
RF^c^	(1) It outputs importance of variable.	(1) No interpretability.
	(2) Individual decision trees can be trained in parallel.	(2) A large number of trees can make the algorithm too slow and ineffective for real-time predictions.
	(3) It reduces overfitting, since RF takes the average value from the outcomes of its constituent decision trees.	(3) Overfitting can occur easily.
	(4) There is no need for feature normalization.	(4) It favors those variables or attributes that can take high number of different values in estimating variable importance.
	(5) Scales well for large datasets.	
	(6) RF is harder to overfit than GBDT.	
GBDT^d^	(1) Often provides predictive accuracy that cannot be beat.	(1) GBDT are more sensitive to overfitting if the data is noisy.
	(2) Lots of flexibility - can optimize on different loss functions and provides several hyperparameter tuning options that make the function fit very flexible.	(2) Training generally takes longer because of the fact that trees are built sequentially.
	(3) No data pre-processing required.	(3) GBDT are harder to tune than RF. There are typically three parameters: number of trees, depth of trees and learning rate, and each tree built is generally shallow.
	(4) Handles missing data, imputation not required.	
	(5) GBDT are better learners than RF.	
SVM^e^	(1) More robust compared to LR	(1) Memory intensive
	(2) Can model non-linear decision boundaries, and there are many kernels to choose from.	(2) A time-consuming approach if applied to a huge database.
	(3) Fairly robust against overfitting, especially in high-dimensional space.	(3) It is trickier to tune due to the importance of picking the right kernel, and don't scale well to larger datasets.
		(4) RF are usually preferred over SVM
DNN^f^	(1) They are usually outperformed by tree ensembles for classical ML problems.	(1) A black box approach for a statistical modeler we have very little control on what the model does.
	(2) Can detect complex nonlinear relationships between dependent and independent variables.	(2) Non-repetitive results and instability.
	(3) Strong gradual corruption ability.	(3) It require much more expertise to tune (i.e., set the architecture and hyperparameters).
	(4) Corruption of one or more cells of ANN^g^ does not prevent it from generating output.	(4) It needs parallel processing environment.

a ML, machine learning;

b LR, logistic regression;

c RF, random forests;

d GBDT, gradient-boosted decision tree;

e SVM, support vector machine;

f DNN, deep neural network;

g ANN, artificial neural network.

### LR

Logistic Regression is a ML algorithm which is used for the classification problems, it is a predictive analysis algorithm and based on the concept of probability (47). We implemented LR algorithm in an R and the used the glm function to fit the model.

### Lasso regression

Lasso regularization automatically deletes unnecessary covariates, and only the most significant variables are retained in the final model. We used 10-fold cross validation to obtain the optimal value of the regularization parameter (lambda) with minimum mean squared error (MSE) (48). The optimal lambda values were used for variable selection. The methods presented above are implemented in an R package called glmnet. minMSE is automatically calculated using arguments s = “lambda.min” in the cv.glmnet function.

### RF

RF is a meta-estimator that fits a number of decision tree classifiers in various sub-samples of the dataset and uses averaging to improve the predictive accuracy and control overfitting (49). In this study, we used out-of-bag estimation to measure the prediction errors. In addition, we used *R* ranger and caret packages to construct RF models.

### GBDT

GBDT produces a prediction model in the form of an ensemble of weak prediction models, builds the model in a stage-wise manner and generalizes them by allowing the optimisation of an arbitrary differentiable loss function (50). In this study, we used 10-fold cross-validation to measure the prediction error and used the XGBoost package in R software to construct GBDT models.

### SVM

SVM is a discriminative classifier that is formally defined by a separating hyperplane. In other words, given labeled training data (supervised learning), the algorithm outputs an optimal hyperplane that categorizes new examples (46). The radial basis function (RBF) kernel in the SVM function was used in this study.

### DNN

DNN is an artificial neural network (ANN) with multiple layers between the input and output layers. DNN searches for the correct mathematical manipulation to turn the input into the output, whether it is a linear relationship or a non-linear one (51). In DNN, we constructed a three-layer feedforward model with an adaptive moment estimation optimiser by utilizing the Keras package. For DNN, we developed the final models by randomly and manually tuning the hyperparameters, such as the number of layers and hidden units, learning rate, learning rate decay, batch size and epochs, by using the Keras package. To minimize potential overfitting, we used batch normalization that normalizes the means and variances of layer inputs.

### Model evaluation

In the test set (30% random sample), we used the AUC value and prospective prediction results [sensitivity (Eq. 1), specificity (Eq. 2), accuracy (Eq. 3), positive predictive value (PPV) (Eq. 4), and negative predictive value (NPV) (Eq. 5)] to evaluate the performance for each ML model. We selected the threshold value of expected prediction results based on receiver operating curve (ROC) (i.e., the value with the shortest distance to the perfect model). A widely used test to compare the difference between two AUCs relies on the method developed in a seminal paper by DeLong et al. (52) (henceforth “the DeLong test”). The DeLong test was applied to compare the differences in the ROC curves of different ML models. Decision curve analysis (DCA) was performed to calculate the clinical “net benefit” for the six ML prediction models in comparison with default strategies of treating all or no patients. Net benefit was calculated across a range of threshold probabilities, which is defined as the minimum probability of a disease at which further intervention would be warranted, as follows: net benefit = sensitivity × prevalence – (1 – specificity) × (1 – prevalence) × *w*, where w is the odds at the threshold probability (53). To obtain an in-depth understanding of the contribution of each predictor to the ML model, we also calculated the importance of variables in the GBDT and RF model for each result.


(1) $$\text{Sensitivity}=\frac{\text{TP}}{\text{TP}+\text{FN}}$$

(2) $$\text{Specificity}=\frac{\text{TN}}{\text{TN}+\text{FP}}$$

(3) $$\text{Accuracy}=\frac{\text{TN}+\text{TP}}{\text{TN}+\text{TP}+\text{FN}+\text{FP}}$$

(4) $$\text{Positive predictive value}=\frac{\text{TP}}{\text{TP}+\text{FP}}$$

(5) $$\text{Negative predictive value}=\frac{\text{TN}}{\text{TN}+\text{FN}}$$

Here, true negatives (TN) and true positives (TP) indicate the elderly that were accurately identified as not suffering depression and suffering depression, respectively; false negatives (FN) and false positives (FP) indicate the elderly that were inaccurately identified as not suffering depression and suffering depression, respectively.

## Results

### Characteristics of elderly samples with CKD in HABCS 2012

As shown in Table 2, amongst the 925 elderly in HABCS 2014, 289 (18.8%) had CKD. The age of the elderly with CKD (87.34 ± 10.73) was higher than that of the elderly without CKD (78.67 ± 10.44). The female elderly with CKD (25.4%) had a larger proportion than the male elderly (12.4%). The marital status of married but not living with spouse (15.2%) for the elderly accounted for a larger proportion than the marital status of married and living with spouse and divorced (9.6 and 9.1%, respectively). The cognitive function score of the elderly with CKD (19.34 ± 5.80) was lower than that of the elderly without CKD (21.01 ± 3.99). The elderly who smoked (12.0%) had a larger proportion than the elderly who did not smoke (20.1%). The ADL/IADL score of the elderly with CKD (36.57 ± 6.79) was lower than that of the elderly without CKD (39.08 ± 5.42). The plasma albumin level of the elderly with CKD (39.04 ± 5.36) was lower than that of those without CKD (41.23 ± 4.51). The total cholesterol level of the elderly with CKD (4.18 ± 0.97) was lower than that of the elderly without CKD (4.36 ± 0.94). The hemoglobin level of the elderly with CKD (126.55 ± 20.61) was lower than that of the elderly without CKD (132.4 ± 22.32). The elderly with hypertension (26.1%) had a larger proportion than the elderly without hypertension (15.8%).

**Table 2:** Characteristics and odds ratio of elderly with depression presenting to the CLHLS 2014.

**Predictors**		**All elderly *N*^a^ (%)**	**Elderly with CKD^b^ *N* (%)**	**Elderly with non-CKD *N* (%)**	***p*** **-value**	**Crude OR^c^ (95%CI^d^)**	**Adjusted OR^g^ (95%CI)**
Age		80.27 ± 11.01	87.34 ± 10.73	78.67 ± 10.44	< 0.001	1.072 (1.056–1.089)	1.066 (1.041–1.092)
Gender	Male	499 (53.9)	62 (12.4)	437 (87.6)	< 0.001	Reference	Reference
	Female	426 (46.1)	108 (25.4)	318 (74.6)		2.394 (1.697–3.377)	1.897 (1.152–3.122)
Marital status	Married and living with spouse	500 (54.1)	48 (9.6)	452 (90.4)	< 0.001	Reference	Reference
	Married but not living with spouse	414 (44.8)	121 (29.2)	293 (70.8)		3.889 (2.699–5.603)	1.837 (1.161–2.907)
	Divorced	11 (1.2)	1 (9.1)	10 (90.9)		0.942 (0.118–7.515)	0.794 (0.082–7.723)
Cognitive function		20.70 ± 4.43	19.34 ± 5.80	21.01 ± 3.99	< 0.001	0.929 (0.899–0.961)	1.010 (0.967–1.056)
Smoking	No	725 (78.4)	146 (20.1)	579 (79.9)	0.009	Reference	Reference
	Yes	200 (21.6)	24 (12.0)	176 (88.0)		0.541 (0.340–0.860)	0.891 (0.496–1.603)
Drinking	No	747 (80.8)	142 (19.0)	605 (81.0)	0.31	Reference	Reference
	Yes	178 (19.2)	28 (15.7)	150 (84.3)		0.795 (0.511–1.239)	1.222 (0.704–2.122)
ADL/IADL^e^		38.62 ± 5.78	36.57 ± 6.79	39.08 ± 5.42	< 0.001	0.939 (0.916–0.963)	1.027 (0.989–1.068)
BMI^f^		22.59 ± 16.10	24.86 ± 35.20	22.08 ± 6.21	0.31	1.009 (0.995–1.022)	1.025 (0.999–1.052)
Waist circumference		82.55 ± 32.10	82.97 ± 11.18	82.45 ± 35.13	0.74	1.000 (0.996–1.005)	1.001 (0.997–1.006)
Plasma albumin		40.83 ± 4.75	39.04 ± 5.36	41.23 ± 4.51	< 0.001	0.906 (0.874–0.940)	0.928 (0.885–0.973)
Blood urea nitrogen		6.29 ± 1.64	6.43 ± 1.75	6.25 ± 1.61	0.23	1.067 (0.965–1.179)	1.133 (1.012–1.267)
Total cholesterol		4.33 ± 0.95	4.18 ± 0.97	4.36 ± 0.94	0.03	0.813 (0.679–0.974)	0.925 (0.727–1.177)
Triglyceride		0.99 ± 0.61	0.97 ± 0.56	1 ± 0.63	0.59	0.931 (0.703–1.234)	1.142 (0.808–1.613)
Urea acid		270.37 ± 79.02	270.21 ± 74.89	270.4 ± 79.97	0.98	1.000 (0.998–1.002)	1.004 (1.001–1.006)
Hemoglobin		131.33 ± 22.12	126.55 ± 20.61	132.4 ± 22.32	0.001	0.987 (0.979–0.995)	0.998 (0.989–1.008)
Hypertension	No	691 (74.7)	109 (15.8)	582 (84.2)	< 0.001	Reference	Reference
	Yes	234 (25.3)	61 (26.1)	173 (73.9)		1.883 (1.318–2.689)	1.957 (1.298–2.951)
Diabetes	No	903 (97.6)	165 (18.3)	738 (81.7)	0.59	Reference	Reference
	Yes	22 (2.4)	5 (22.7)	17 (77.3)		1.316 (0.479–3.617)	1.736 (0.556–5.416)
Heart disease	No	853 (92.2)	154 (18.1)	699 (81.9)	0.38	Reference	Reference
	Yes	72 (7.8)	16 (22.2)	56 (77.8)		1.297 (0.724–2.322)	1.068 (0.541–2.108)
Stroke and cerebrovascular diseases	No	871 (94.2)	160 (18.4)	711 (81.6)	0.98	Reference	Reference
	Yes	54 (5.8)	10 (18.5)	44 (81.5)		1.010 (0.498–2.050)	0.567 (0.252–1.273)
Cancer	No	922 (99.7)	169 (18.3)	753 (81.7)	0.50	Reference	Reference
	Yes	3 (0.3)	1 (33.3)	2 (66.7)		2.228 (0.201–24.711)	2.232 (0.162–30.816)
Blood disease	No	913 (98.7)	168 (18.4)	745 (81.6)	0.88	Reference	Reference
	Yes	12 (1.3)	2 (16.7)	10 (83.3)		0.887 (0.193–4.085)	1.511 (0.279–8.191)
Depression	No	869 (93.9)	160 (18.4)	709 (81.6)	0.92	Reference	Reference
	Yes	56 (6.1)	10 (17.9)	46 (82.1)		0.963 (0.476–1.950)	1.024 (0.468–2.242)

a N represents the samples;

b CKD, chronic kidney disease;

c OR, odds ratio;

d CI, confidence interval;

e ADL/IADL, activities of daily living/instrumental activities of daily living;

f BMI, body mass index.

g An adjusted OR is an odds ratio that controls for other predictor variables in the binary logistic regression.

Afterward, we performed binary logistic regression to analyze the crude and adjusted odds ratios for the elderly with CKD (vs. non-CKD) for each predictor. The elderly with a high age had a higher risk of CKD compared with the elderly with a low age [adjusted odds ratio (OR) = 1.066; 95% confidence interval (CI): 1.041–1.092]. The female elderly had a higher risk of CKD than the male elderly (adjusted OR = 1.897; 95% CI: 1.152–3.122). The elderly with a marital status of married but not living with spouse had larger odds of CKD than the elderly with a marital status of married and living with spouse (adjusted OR = 1.837; 95% CI: 1.161–2.907). The elderly with low plasma albumin had a higher risk of CKD than the elderly with high plasma albumin (adjusted OR = 0.928; 95% CI: 0.885–0.973). The elderly with high urea acid had a higher risk of CKD than those with low urea acid (adjusted OR = 1.004; 95% CI: 1.001–1.006). The analysis showed that the elderly with hypertension (adjusted OR=1.957; 95%CI: 1.298–2.951) had a higher risk of CKD than those without hypertension.

### Prediction appearance for elderly with CKD by using the six ML models

**Figure 2:**
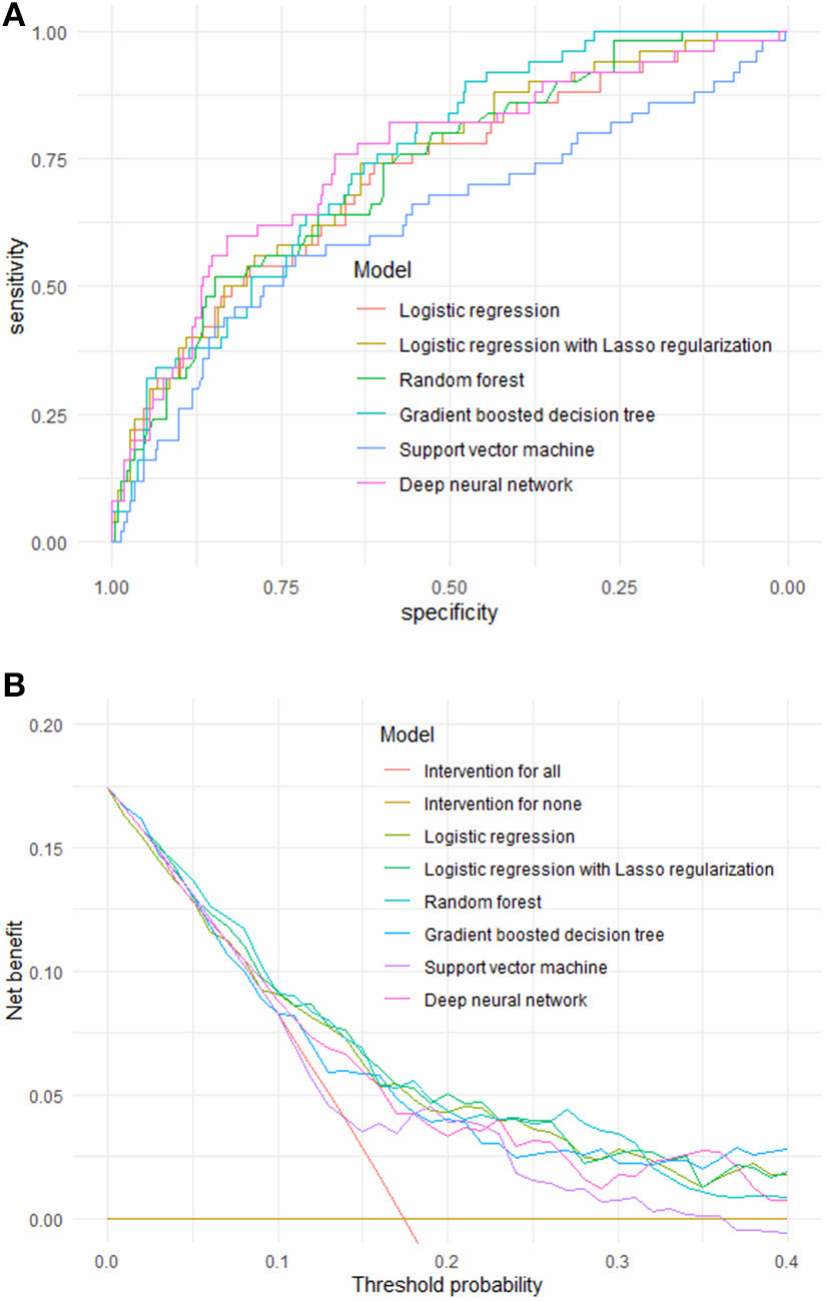
Predictive performance of six machine learning models for elderly with CKD **(A)** ROC curve. The x-axis represents specificity (probability of negative test given that the elderly did not have the CKD), and the y-axis represents sensitivity (probability of a positive test given that the elderly had the CKD). **(B)** Decision curve analysis. The y-axis is benefit and the x-axis is preference. The benefit of a test or model is that it correctly identifies which patients do and do not have disease (in our example, CKD).

A comparison of the ROC curves of the six ML models for elderly with CKD is shown in Figure 2A and Table 3. We further compared the models by using the DeLong test. LR, lasso regression, RF, GBDT and DNN showed no statistical significance (AUC > 0.7), implying that these models had similar predictive power. In our study, SVM had the lowest predictive performance (AUC = 0.633, *p*-value = 0.06). The threshold values of LR, lasso regression, RF, GBDT, SVM, and DNN were 0.127, 0.138, 0.272, 0.050, 0.180, and 0.352, respectively. SVM had the highest accuracy (0.732, 95% CI: [0.677–0.782]), and LR had the lowest accuracy (0.634, 95% CI: [0.576–0.690]). SVM had the lowest sensitivity (0.460), and DNN had the highest sensitivity (0.760). The specificity of SVM was the highest (0.716), and the specificity of the LR was the lowest (0.612). DNN had the highest PPV (0.328), whereas LR had the lowest PPV (0.287). DNN had the highest NPV (0.930), whereas SVM had the lowest NPV (0.874). DCA (Figure 2B) showed that within the threshold ranges of ~0–0.03 and 0.37–0.40, the net benefit of GBDT was the largest, and within the threshold ranges of ~0.03–0.10 and 0.26–0.30, the net benefit of RF was the largest.

**Table 3:** Prediction performance of elderly with CKD using 6 machine learning models.

**Model**	**AUC^a^**	***p*** **-value^d^**	**Threshold**	**Accuracy**	**Sensitivity**	**Specificity**	**PPV^b^**	**NPV^c^**
Logistic regression	0.716	Reference	0.127	0.634 (0.576–0.690)	0.740	0.612	0.287	0.918
Logistic regression with Lasso regularization	0.734	0.28	0.138	0.652 (0.593–0.707)	0.740	0.633	0.298	0.920
Random forest	0.729	0.71	0.272	0.686 (0.629–0.740)	0.640	0.696	0.308	0.902
Gradient boosted decision tree	0.750	0.34	0.050	0.659 (0.601–0.713)	0.720	0.646	0.300	0.916
Support vector machine	0.633	0.06	0.180	0.732 (0.677–0.782)	0.460	0.789	0.315	0.874
Deep neural network	0.750	0.56	0.352	0.686 (0.629–0.740)	0.760	0.671	0.328	0.930

a AUC, area under the receiver operating curve;

b PPV, positive predictive value;

c NPV, negative predictive value.

d p-value is the result of Delong test of AUC curve based on the comparison of each machine learning model.

### Importance of CKD predictors for the elderlys

**Figure 3:**
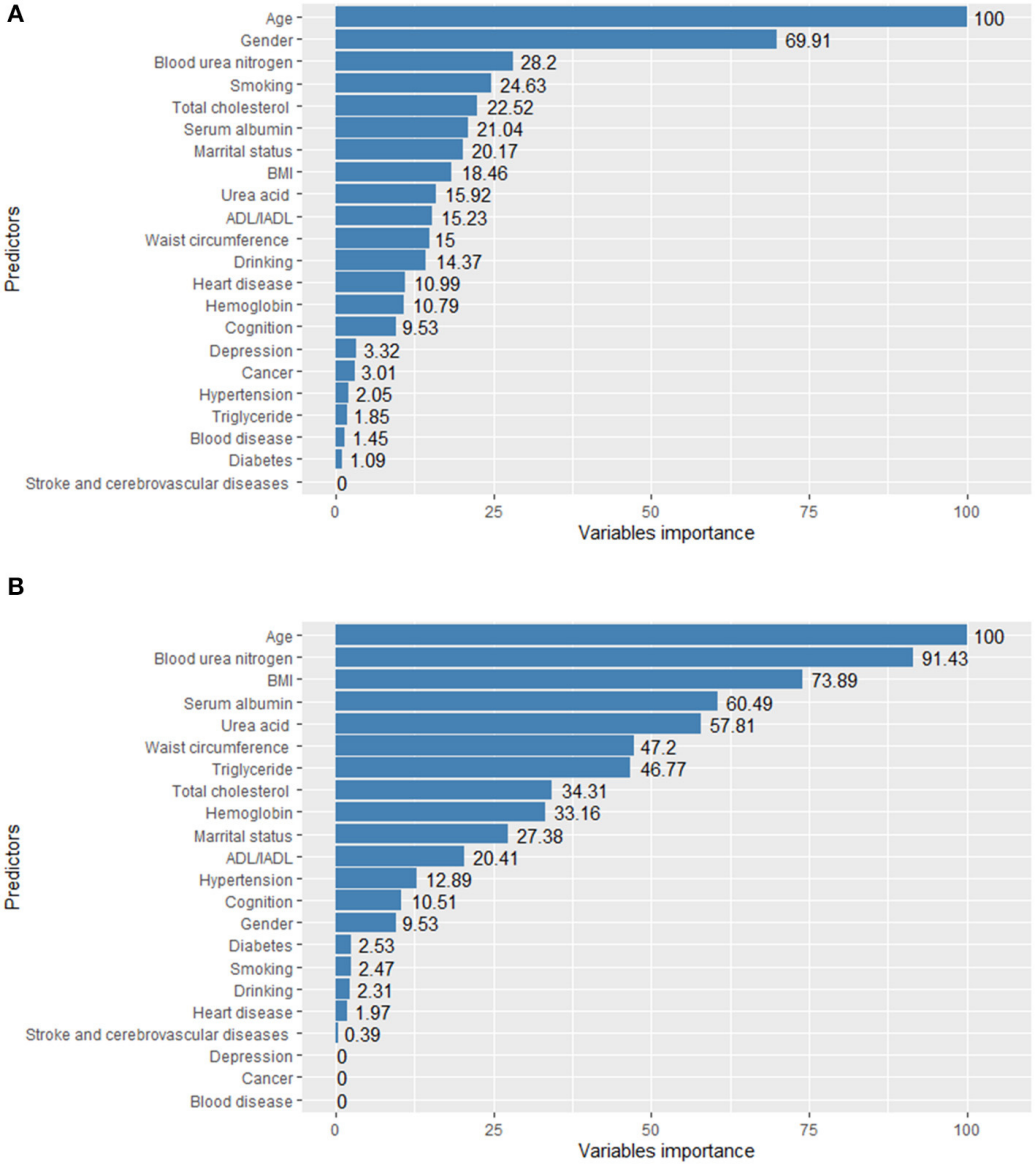
Variable importance of RF **(A)** and GBDT **(B)** model for elderly with CKD.

The importance of the predictors in the RF and GBDT models is shown in Figure 3. Age was the most important predictor variable in both models. In addition, blood urea nitrogen, serum albumin, uric acid, BMI, marital status, ADL/IADL and gender were crucial in predicting CKD amongst the elderly.

## Discussion

This study was based on the panel data of 925 elderly individuals in the 2012 baseline survey and 2014 follow-up survey of the HABCS database. The data included the socioeconomic status, unhealthy lifestyle, chronic diseases and other biological indicators of each elderly person. We used six ML models (LR, lasso regression, RF, GBDT, SVM, and DNN) to predict the risk of CKD in the elderly 2 years later. The results showed that the ML model has excellent performance in predicting the risk of CKD in the elderly. The DCA result indicated that all ML models can generate a huge net benefit within various thresholds, and each ML model has its own advantages derived from its net benefit within different threshold ranges.

This study found that the elderly with old age, female, married but not living with a spouse, low plasma albumin, high urea acid and hypertension have a high risk of CKD, which is consistent with the results of several studies (1, 54, 55). Previous studies on CKD risk prediction did not analyze the elderly individually (56–58). The age of respondents in several previous studies was 40 or over 50, but according to the prediction results of the ML model, the AUC value of these studies are all above 0.7. The AUC value of the ML model in this study was between 0.716 and 0.750, which is effective in predicting CKD in the elderly.

Several studies have shown that the ML prediction model is driven by automatic prediction based on the most objective indicators (59, 60). It can use the complex nonlinear relationship between predictors to improve prediction performance. When the sample size is sufficient, the predictive performance of the ML algorithm is good. However, in certain sample screening procedures, the sample size is insufficient. Therefore, we must ensure that the ML algorithm can obtain good prediction performance with a small sample size. In this study, although we selected a relatively small data set of 925 elderly people, the sample size meets the requirements of power analysis and can be used to perform high-precision prediction tasks. The AUC value of most models is close to 0.7, and the performance of linear models is better than that of other types of models. In this study, due to the small amount of data, the linear classifiers could separate samples ideally, and the highly complex ML models (e.g., SVM) demonstrated powerful learning capabilities but were prone to overfitting. The forecast accuracy was thus reduced. SVM performed the worst in this study because the Euclidean distance on which SVM relies is not the best way to deal with the classification of CKD in the elderly. Therefore, the linear model performed better in our study.

When predicting the risk of CKD in the elderly 2 years later, we should determine a threshold for identifying CKD and non-CKD in the elderly. If a model has high PPV and NPV, then it is theoretically ideal. However, in practice, we need to weigh high PPV and high NPV based on the actual situation. In this study, because the proportion of elderly people suffering from CKD is small, we needed to consider the PPV results of different models. This task allows researchers to measure the prediction of the model so that many potential high-risk CKD groups can be screened out. In addition, we used DCA to analyse the net benefits of six ML models at different thresholds. The results can help medical service providers provide flexible model selections based on their professional knowledge to guide clinical decision-making.

This is the first survey to comprehensively study the practicality of different modern ML models in predicting CKD in the elderly in China. The decision support system based on the predictive model in this research can help medical staff detect and intervene in the health of the elderly early, and it can provide scientific evidence for clinical treatment, disease prevention and community health management.

Our study has several limitations. Firstly, only 22 predictors were considered. We restricted our analyses to predictive modeling with known or possible risk factors, including demographic indicators, biomarkers of kidney function and kidney damage, such as ethnicity, serum cystatin C and renalase. Hence, our conclusion cannot be generalized to data sets with numerous predictors. In future studies, we can continue to add more specific predictors to help improve the prediction performance, such as the size and cortical thickness of the kidney. Secondly, the diagnostic criteria for chronic diseases were mainly based on self-reported medical history, and related variables were obtained through questionnaire surveys. No clear test diagnosis was performed. Thus, deviations may be present. Thirdly, the sample size used was relatively small; the tuning parameters could be optimized further to avoid overfitting. In follow-up research, we can consider recruiting more participants for model testing and evaluation. Lastly, the capability to diagnose depression amongst the elderly depends on local medical resources, and indications and clinical thresholds may vary between emergency departments and clinicians.

## Conclusion

We established and compared six ML models that can predict the risk of CKD 2 years later based on the socioeconomic characteristics, unhealthy lifestyle, chronic diseases and biomedical indicators of the elderly. The LR, lasso, RF and ML models, including GBDT and DNN, demonstrated a high overall predictive capability, and the different models showed high net benefit at different threshold levels. We also found that age, blood urea nitrogen, serum albumin, uric acid, BMI, marital status and ADL/IADL exerted an important influence on model predictability, whereas the other predictors were not as important. Further research is required to test the effect of using the system in a clinical environment.

## Data availability statement

Publicly available datasets were analyzed in this study. This data can be found here: https://opendata.pku.edu.cn/dataset.xhtml?persistentId=10.18170/DVN/WBO7LK.

## Ethics statement

The studies involving human participants were reviewed and approved by Ethics Committee of Peking University (IRB00001052-13074). The patients/participants provided their written informed consent to participate in this study.

## Author contributions

DS and NW are the guarantors and contributed to the conception and design of the project. DS, KH, and XZ contributed to the analysis and interpretation of the data. DS, XZ, and YC contributed to the data acquisition and provided statistical analysis support. DS drafted the article. The corresponding author attests that all listed authors meet authorship criteria and that no others meeting the criteria have been omitted.

## Funding

This study was supported by Michigan Institute for Clinical and Health Research (MICHR No. UL1TR002240) and National Natural Science Foundation of China (No. 71974134).

## Conflict of interest

The authors declare that the research was conducted in the absence of any commercial or financial relationships that could be construed as a potential conflict of interest.

## Publisher's note

All claims expressed in this article are solely those of the authors and do not necessarily represent those of their affiliated organizations, or those of the publisher, the editors and the reviewers. Any product that may be evaluated in this article, or claim that may be made by its manufacturer, is not guaranteed or endorsed by the publisher.

## References

[1] Zhang L Wang F Wang L Wang W Liu B Liu J. Prevalence of chronic kidney disease in China: a cross-sectional survey. Lancet. (2012) 379:815–22. 10.1016/S0140-6736(12)60033-6 22386035

[2] Bikbov B Purcell CA Levey AS Smith M Abdoli A Abebe M. Global, regional, and national burden of chronic kidney disease, 1990–2017: a systematic analysis for the Global Burden of Disease Study 2017. Lancet. (2020) 395:709–33. 10.1016/S0140-6736(20)30045-3 32061315 PMC7049905

[3] Keith DS Nichols GA Gullion CM Brown JB Smith DH. Longitudinal follow-up and outcomes among a population with chronic kidney disease in a large managed care organization. Arch Internal Med. (2004) 164:659–63. 10.1001/archinte.164.6.659 15037495

[4] Go AS Chertow GM Fan D McCulloch CE Hsu CY. Chronic kidney disease and the risks of death, cardiovascular events, and hospitalization. N Engl J Med. (2004) 351:1296–305. 10.1056/NEJMoa041031 15385656

[5] Smyth A Glynn LG Murphy AW Mulqueen J Canavan M Reddan DN. Mild chronic kidney disease and functional impairment in community-dwelling older adults. Age Ageing. (2013) 42:488–94. 10.1093/ageing/aft007 23438445

[6] Tamhane U Voytas J Aboufakher R. Maddens M. Do hemoglobin and creatinine clearance affect hospital readmission rates from a skilled nursing facility heart failure rehabilitation unit? J Am Med Direct Assoc. (2008) 9:194–8. 10.1016/j.jamda.2007.12.004 18294603

[7] Wang F Yang C Long J Zhao X Tang W Zhang D. Executive summary for the 2015 annual data report of the China kidney disease network (CK-NET). Kidney Int. (2019) 95:501–5. 10.1016/j.kint.2018.11.011 30784660

[8] Stevens PE. O'donoghue DJ De Lusignan S Van Vlymen J Klebe B Middleton R. Chronic kidney disease management in the United Kingdom: NEOERICA project results. Kidney Int. (2007) 72:92–9. 10.1038/sj.ki.5002273 17440495

[9] Liu ZH. Nephrology in China. Nat Rev Nephrol. (2013) 9:523. 10.1038/nrneph.2013.146 23877587

[10] Carrero JJ Hecking M Chesnaye NC Jager KJ. Sex and gender disparities in the epidemiology and outcomes of chronic kidney disease. Nat Rev Nephrol. (2018) 14:151. 10.1038/nrneph.2017.181 29355169

[11] Hill NR Fatoba ST Oke JL Hirst JA O'Callaghan CA Lasserson DS. Global prevalence of chronic kidney disease–a systematic review and meta-analysis. PLoS ONE. (2016) 11:e0158765. 10.1371/journal.pone.0158765 27383068 PMC4934905

[12] Huda MN Alam KS. Prevalence of chronic kidney disease and its association with risk factors in disadvantageous population. Int J Nephrol. (2012) 2012:267329. 10.1155/2012/267329 22848823 PMC3400350

[13] Xia J Wang L Ma Z Zhong L Wang Y Gao Y. Cigarette smoking and chronic kidney disease in the general population: a systematic review and meta-analysis of prospective cohort studies. Nephrol Dial Transpl. (2017) 32:475–87. 10.1093/ndt/gfw452 28339863

[14] Lai YJ Chen YY Lin YK Chen CC Yen YF Deng CY. Alcohol consumption and risk of chronic kidney disease: a nationwide observational cohort study. Nutrients. (2019) 11:2121. 10.3390/nu11092121 31489891 PMC6769971

[15] Viscogliosi G De Nicola L Vanuzzo D Giampaoli S Palmieri L Donfrancesco C. Mild to moderate chronic kidney disease and functional disability in community-dwelling older adults. The cardiovascular risk profile in renal patients of the Italian health examination survey (CARHES) study. Arch Gerontol Geriat. (2019) 80:46–52. 10.1016/j.archger.2018.10.001 30343147

[16] Anand S Johansen KL Kurella Tamura M. Aging and chronic kidney disease: the impact on physical function and cognition. J Gerontol Ser A Biomed Sci Med Sci. (2014) 69:315–22. 10.1093/gerona/glt109 23913934 PMC4017829

[17] Novak M Mucsi I Rhee CM Streja E Lu JL Kalantar-Zadeh K. Increased risk of incident chronic kidney disease, cardiovascular disease, and mortality in patients with diabetes with comorbid depression. Diabetes Care. (2016) 39:1940–7. 10.2337/dc16-0048 27311494 PMC5079613

[18] Herrington WG Smith M Bankhead C Matsushita K Stevens S Holt T. Body-mass index and risk of advanced chronic kidney disease: prospective analyses from a primary care cohort of 14 million adults in England. PLoS ONE. (2017) 12:e0173515. 10.1371/journal.pone.0173515 28273171 PMC5342319

[19] He Y Li F Wang F Ma X Zhao X Zeng Q. The association of chronic kidney disease and waist circumference and waist-to-height ratio in Chinese urban adults. Medicine. (2016) 95:3769. 10.1097/MD.0000000000003769 27336864 PMC4998302

[20] Anupama YJ Hegde SN Uma G Patil M. Hypertension is an important risk determinant for chronic kidney disease: results from a cross-sectional, observational study from a rural population in South India. J Hum Hypert. (2017) 31:327–32. 10.1038/jhh.2016.81 27882930

[21] Chang HL Wu CC Lee SP Chen YK Su W Su SL. predictive model for progression of CKD. Medicine. (2019) 98:e16186. 10.1097/MD.0000000000016186 31261555 PMC6617424

[22] Bansal N Matheny ME. Greevy RA Eden SK Perkins AM Parr SK. Acute kidney injury and risk of incident heart failure among US veterans. Am J Kidney Dis. (2018) 71:236–45. 10.1053/j.ajkd.2017.08.027 29162339

[23] Menon V Gul A Sarnak MJ. Cardiovascular risk factors in chronic kidney disease. Kidney Int. (2005) 68:1413–8. 10.1111/j.1523-1755.2005.00551.x 16164615

[24] Chen DP Davis BR Simpson LM Cushman WC Cutler JA Dobre M. Association between chronic kidney disease and cancer mortality: a report from the ALLHAT. Clin Nephrol. (2017) 87:11. 10.5414/CN108949 27900942 PMC13055424

[25] Lang J Katz R Ix JH Gutierrez OM Peralta CA Parikh CR. Association of serum albumin levels with kidney function decline and incident chronic kidney disease in elders. Nephrol Dial Transpl. (2018) 33:986–92. 10.1093/ndt/gfx229 28992097 PMC6251663

[26] Tu H Wen CP Tsai SP Chow WH Wen C Ye Y. Cancer risk associated with chronic diseases and disease markers: prospective cohort study. BMJ. (2018) 360:k134. 10.1136/bmj.k134 29386192 PMC5791146

[27] Dincer N Dagel T Afsar B Covic A Ortiz A Kanbay M. The effect of chronic kidney disease on lipid metabolism. Int Urol Nephrol. (2019) 51:265–77. 10.1007/s11255-018-2047-y 30519980

[28] Zhang YF He F Ding HH Dai W Zhang Q Luan H. Effect of uric-acid-lowering therapy on progression of chronic kidney disease: a meta-analysis. J Huazhong Univ Sci Technol Med Sci. (2014) 34:476–81. 10.1007/s11596-014-1302-4 25135714

[29] Xu L Chen Y Xie Z He Q Chen S Wang W. High hemoglobin is associated with increased in-hospital death in patients with chronic obstructive pulmonary disease and chronic kidney disease: a retrospective multicenter population-based study. BMC Pulmon Med. (2019) 19:1–8. 10.1186/s12890-019-0933-4 31533673 PMC6749661

[30] Perotte A Ranganath R Hirsch JS Blei D Elhadad N. Risk prediction for chronic kidney disease progression using heterogeneous electronic health record data and time series analysis. J Am Med Inform Assoc. (2015) 22:872–80. 10.1093/jamia/ocv024 25896647 PMC4482276

[31] Echouffo-Tcheugui JB Kengne AP. Risk models to predict chronic kidney disease and its progression: a systematic review. PLoS Med. (2012) 9:e1001344. 10.1371/journal.pmed.1001344 23185136 PMC3502517

[32] Tangri N Kitsios GD Inker LA Griffith J Naimark DM Walker S. Risk prediction models for patients with chronic kidney disease: a systematic review. Ann Internal Med. (2013) 158:596–603. 10.7326/0003-4819-158-8-201304160-00004 23588748

[33] Liu R Li X Zhang W Zhou HH. Comparison of nine statistical model based warfarin pharmacogenetic dosing algorithms using the racially diverse international warfarin pharmacogenetic consortium cohort database. PLoS ONE. (2015) 10:e0135784. 10.1371/journal.pone.0135784 26305568 PMC4549222

[34] Orru G Pettersson-Yeo W Marquand AF Sartori G Mechelli A. Using support vector machine to identify imaging biomarkers of neurological and psychiatric disease: a critical review. Neurosci Biobehav Rev. (2012) 36:1140–52. 10.1016/j.neubiorev.2012.01.004 22305994

[35] Zhang X Bellolio MF Medrano-Gracia P Werys K Yang S Mahajan P. Use of natural language processing to improve predictive models for imaging utilization in children presenting to the emergency department. BMC Med Inform Dec Making. (2019) 19:287. 10.1186/s12911-019-1006-6 31888609 PMC6937987

[36] Lee Y Ragguett RM Mansur RB Boutilier JJ Rosenblat JD Trevizol A. Applications of machine learning algorithms to predict therapeutic outcomes in depression: a meta-analysis and systematic review. J Affect Disord. (2018) 241:519–32. 10.1016/j.jad.2018.08.073 30153635

[37] Cosgun E Limdi NA Duarte CW. High-dimensional pharmacogenetic prediction of a continuous trait using machine learning techniques with application to warfarin dose prediction in African Americans. Bioinformatics. (2011) 27:1384–9. 10.1093/bioinformatics/btr159 21450715 PMC3087957

[38] Mark E Goldsman D Gurbaxani B Keskinocak P Sokol J. Using machine learning and an ensemble of methods to predict kidney transplant survival. PLoS ONE. (2019) 14:e0209068. 10.1371/journal.pone.0209068 30625130 PMC6326487

[39] Zhang X Kim J Patzer RE Pitts SR Patzer A Schrager JD. Prediction of emergency department hospital admission based on natural language processing and neural networks. Methods Inf Med. (2017) 56:377–89. 10.3414/ME17-01-0024 28816338

[40] Vougas K Sakellaropoulos T Kotsinas A Foukas GRP Ntargaras A Koinis F. Machine learning and data mining frameworks for predicting drug response in cancer: an overview and a novel in silico screening process based on association rule mining. Pharmacol Therapeut. (2019) 203:107395. 10.1016/j.pharmthera.2019.107395 31374225

[41] Yin ZX Wang JL Lyu YB Luo JS Zeng Y Shi XM. Association between serum albumin and cognitive performance in elderly Chinese. Zhonghua Liuxingbingxue Zazhi. (2016) 37:1323–6. 10.3760/cma.j.issn.0254-6450.2016.10.001 27765118

[42] Abecassis M Bartlett ST Collins AJ Davis CL Delmonico FL Friedewald JJ. Kidney transplantation as primary therapy for end-stage renal disease: a national kidney foundation/kidney disease outcomes quality initiative (NKF/KDOQI™) conference. Clin J Am Soc Nephrol. (2008) 3:471–80. 10.2215/CJN.05021107 18256371 PMC2390948

[43] Huang YP Zheng T Zhang DH Chen LY Mao PJ. Community-based study on elderly CKD subjects and the associated risk factors. Renal Fail. (2016) 38:1672–6. 10.1080/0886022X.2016.1229987 27758130

[44] Lawton MP Brody EM. Assessment of older people: self-maintaining and instrumental activities of daily living. Gerontologist. (1969) 9(3_Part_1):179–86. 5349366

[45] Katz S Ford AB Moskowitz RW Jackson BA Jaffe MW. Studies of illness in the aged: the index of ADL: a standardized measure of biological and psychosocial function. JAMA. (1963) 185:914–9. 14044222 10.1001/jama.1963.03060120024016

[46] Uddin S Khan A Hossain ME Moni MA. Comparing different supervised machine learning algorithms for disease prediction. BMC Med Inform Dec Making. (2019) 19:1–16. 10.1186/s12911-019-1004-8 31864346 PMC6925840

[47] Dinh A Miertschin S Young A Mohanty SD. A data-driven approach to predicting diabetes and cardiovascular disease with machine learning. BMC Med Inform Dec Making. (2019) 19:1–15. 10.1186/s12911-019-0918-5 31694707 PMC6836338

[48] Waldmann P Mészáros G Gredler B Fuerst C Sölkner J. Evaluation of the lasso and the elastic net in genome-wide association studies. Front Genet. (2013) 4:270. 10.3389/fgene.2013.00270 24363662 PMC3850240

[49] Shreyas R Akshata DM Mahanand BS Shagun B Abhishek CM. Predicting popularity of online articles using random forest regression. In: Proceedings of the 2016 Second International Conference on Cognitive Computing and Information Processing (CCIP). Mysuru: IEEE (2016). 10.1109/CCIP.2016.7802890

[50] Zhou C Yu H Ding Y Guo F Gong XJ. Multi-scale encoding of amino acid sequences for predicting protein interactions using gradient boosting decision tree. PLoS ONE. (2017) 12:e0181426. 10.1371/journal.pone.0181426 28792503 PMC5549711

[51] Kriegeskorte N Golan T. Neural network models and deep learning. Curr Biol. (2019) 29:R231–6. 10.1016/j.cub.2019.02.034 30939301

[52] DeLong ER DeLong DM. Comparing the areas under two or more correlated receiver operating characteristic curves: a nonparametric approach. Biometrics. (1988) 29:837–45. 3203132

[53] Vickers AJ van Calster B Steyerberg EW A. simple, step-by-step guide to interpreting decision curve analysis. Diagnost Prognost Res. (2019) 3:1–8. 10.1186/s41512-019-0064-7 31592444 PMC6777022

[54] Kaze FF Kengne AP Magatsing CT Halle MP Yiagnigni E Ngu KB. Prevalence and determinants of chronic kidney disease among hypertensive Cameroonians according to three common estimators of the glomerular filtration rate. J Clin Hypert. (2016) 18:408–14. 10.1111/jch.12781 26791352 PMC8031998

[55] Soriano S Gonzalez L Martin-Malo A Rodriguez M Aljama P. C-reactive protein and low albumin are predictors of morbidity and cardiovascular events in chronic kidney disease (CKD) 3-5 patients. Clin Nephrol. (2007) 67:352–7. 10.5414/CNP67352 17598370

[56] Xiao J Ding R Xu X Guan H Feng X Sun T. Comparison and development of machine learning tools in the prediction of chronic kidney disease progression. J Transl Med. (2019) 17:119. 10.1186/s12967-019-1860-0 30971285 PMC6458616

[57] Almansour NA Syed HF Khayat NR Altheeb RK Juri RE Alhiyafi J. Neural network and support vector machine for the prediction of chronic kidney disease: a comparative study. Comput Biol Med. (2019) 109:101–11. 10.1016/j.compbiomed.2019.04.017 31054385

[58] Shih CC Lu CJ Chen GD Chang CC. Risk prediction for early chronic kidney disease: results from an adult health examination program of 19,270 individuals. Int J Environ Res Public Health. (2020) 17:4973. 10.3390/ijerph17144973 32664271 PMC7399976

[59] Chong SL Liu N Barbier S Ong MEH. Predictive modeling in pediatric traumatic brain injury using machine learning. BMC Med Res Methodol. (2015) 15:22. 10.1186/s12874-015-0015-0 25886156 PMC4374377

[60] Wellner B Grand J Canzone E Coarr M Brady PW Simmons J. Predicting unplanned transfers to the intensive care unit: a machine learning approach leveraging diverse clinical elements. JMIR Med Inform. (2017) 5:e45. 10.2196/medinform.8680 29167089 PMC5719228

